# Tongue cancer in young patients: case report of a 26-year-old patient

**DOI:** 10.1186/1758-3284-4-20

**Published:** 2012-05-14

**Authors:** Aleksandra Credé, Michael Locher, Marius Bredell

**Affiliations:** 1 University Hospital Zurich, Plattenstrasse 15, CH-8032, Zürich, Switzerland

**Keywords:** Squamous cell carcinoma, SCC, Acute myeloid leukemia, AML, Second malignant neoplasms, Second primary cancers, Childhood cancer

## Abstract

**Introduction:**

This article presents the case of a 26-year-old woman with tongue cancer. The median age at the diagnosis of the tongue’s cancer is 61 years. Only approximately 2% of patients are diagnosed before the age of 35.

**Case presentation:**

Our patient survived acute myeloid leukemia (AML) before her second year. She had been having recurrent, poorly healing aphtae on the right side of the tongue for a period of months before the symptoms of the tongue cancer appeared. As a treatment a partial glossectomy was conducted on the right side and a neck dissection of levels I-III. Than a reconstruction of the tongue with a radialis free vascularised flap from left side was performed.

**Discussion:**

It should be always looked for the causal factor in young patients with a neoplasm. There is strong evidence for second malignant neoplasms in survivors of childhood cancer.

## Background

The morbidity of malignant tumors in young patients is low (only approximately 2 percent of patients diagnosed with tongue cancer are below the age of 35). This was the case of this patient who suffered from an acute myeloid leukemia before she turned two. It is known that survivors of childhood cancer are at risk of developing a second malignant neoplasm.

## Case presentation

### Introduction

Squamous cell carcinoma (SCC) of the tongue is the most frequent intra oral head and neck cancer. European statistics indicate an incidence of around 10–20 per 100 000 of the population. In Western Europe a decrease has been seen in males, contrary to the increase in female subjects that has been evident over the last decade. Many other world regions are finding an general increase of the incidence of oral cancer. [1] The median age at the diagnosis of the tongue’s cancer is 61 years. Only approximately 2% of patients are diagnosed before the age of 35 and another 7% before the age of 45, this despite the fact that there is an increasing trend in the prevalence of tongue SCC. [2-4].

In the literature only three studies have evaluated oral tongue SCC in patients younger than 30 years old. The study published by Soudry et al. [4] in 2010 as well as two further studies [5,6] were done nearly 30 years ago.

Apart from the possibility of a potential genetic tendency there are some well known carcinogens in head and neck squamous cell carcinoma (HNSCC) such as tobacco, alcohol, viruses like the human papilloma virus (HPV), herpes simplex virus (HSV), Epstein-Barr virus (EBV), as well as immunosuppression, polymorphism of interleukin 6 (IL6) and tumor necrosis factor (TNF) [7] and inherited syndromes, e.g. Fanconi anemia, Aplastic anemia [8] that all may play a role in the carcinogenesis process. These factors may be present as single factors, but may in combination play a more compelling role in the early development of HNSCC.

As for other intra oral sites SSC of the tongue is often associated with other potentially malignant or premalignant lesions and conditions such a leukoplakia, erosive lichen planus as well as atrophic glossitis. Poor oral hygiene is also reported to play a role. [9-11].

In young patients the main causal factor of oropharyngeal SCC appears to be HPV infection, associated mostly with cancer of the tonsils. [12] Marijuana remains another possible contributing factor and it would seem that anemia is a co-factor, not only regarding the development, but also regarding prognosis. In contrary to their older counterparts young patients with SCC rather tend to be non-smokers, non-drinkers. [13-15] Patients younger than 30 years exhibit a significantly increased chromosome fragility compared to other patients following exposure to mutagen, [16] which may explain why environmental exposure to smoke probably plays a role in non-smokers.

A separate risk group consists of patients with a second primary cancers in survivors of childhood cancer. It is known that cancer patients have some risk of second synchronous or metachronous primary tumor. [16-19].

### Case report

A 26 year-old patient presented herself at the Oral Surgery Division of the University Hospital Zurich with a main complaint of a tongue lesion.

For the last 4 months she observed an alteration on the right side of the tongue, displaying alarming growth over a three week period. The lesion was painful when the patient moved her tongue or while eating. She further mentioned also having recurrent, poorly healing aphtae on the right side of the tongue for a period of months before the current symptoms presented.

Regarding her medical history she suffered of acute myeloid leukemia (AML) before the age of 2 and 8 years ago she had an incident of a cerebral venous sinus thrombosis. Currently she suffers from epilepsy, but she hasn’t had any symptoms for a long time. She has no known allergies, takes no medicine, no antiepileptic drugs, has been smoking for 8 years 2–3 cigarettes per day and she doesn’t drink alcohol. No weight changes or night sweats were noted.

On examination the patient showed a symmetric face and normal skin color, motor and sensory cranial nerve functions were within normal range. No lymph nodes were palpable in her neck on both sides.

**Figure 1:**
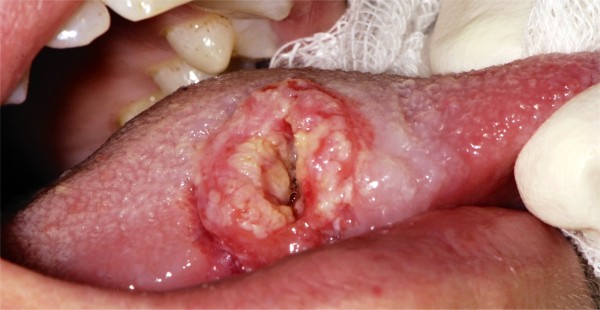
Lateral view of 2 cm ulcer on middle third of the right side of the tongue.

**Figure 2:**
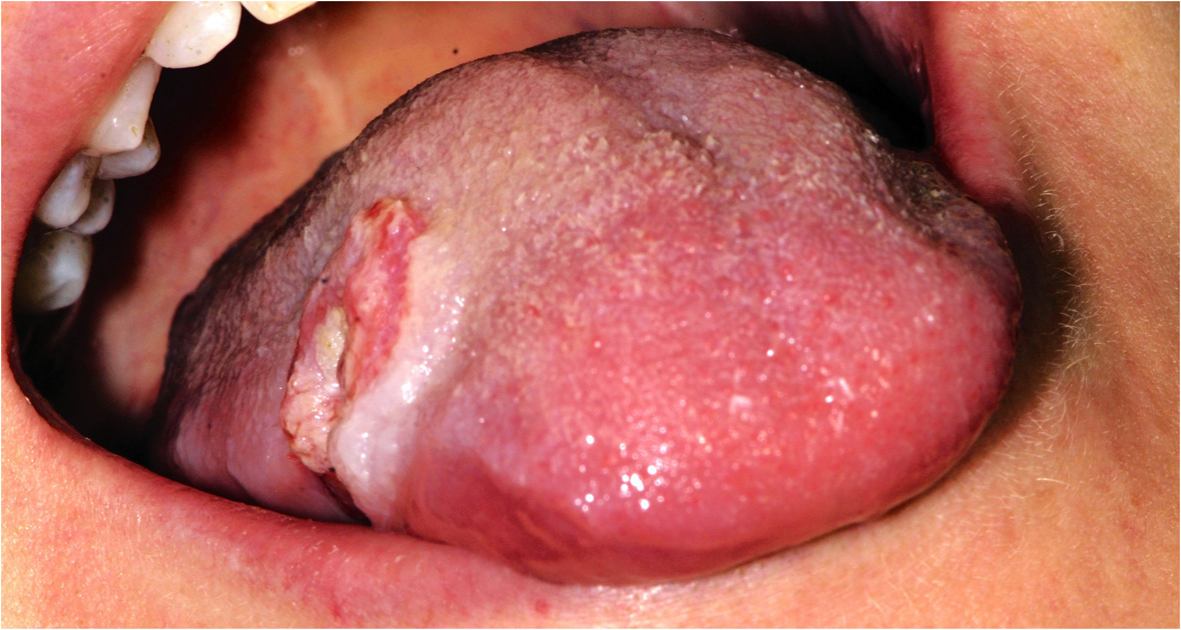
Frontal view of 2 cm ulcer on middle third of the right side of the tongue.

On intra oral examination we found a tumor in the middle third position of the right side of the tongue. The size of the tongue lesion was about 15x20x15 mm (width, length, depth), appearing to be exophytic, with a central ulcer appearing to infiltrate the tongue musculature that appeared to be relatively well demarcated (Figures 1 and 2). The oral mucosa showed signs of leukoedema and maceration on the right buccal mucosa and on the lower lip due to a self inflicted chewing habit. No limitation of the mouth opening was observed and a normal dentition was apparent.

**Figure 3:**
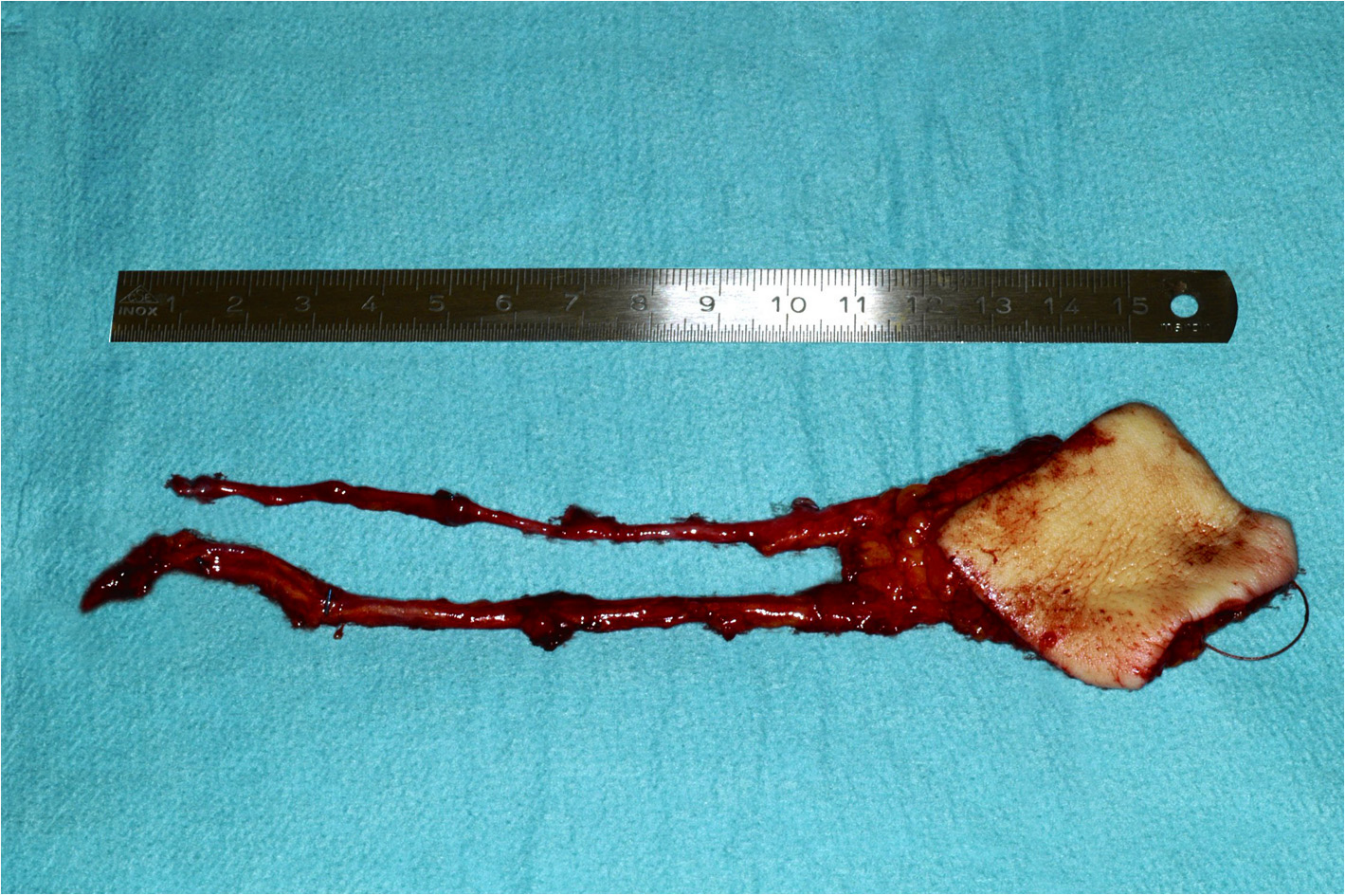
Completed right sided neck dissection.

**Figure 4:**
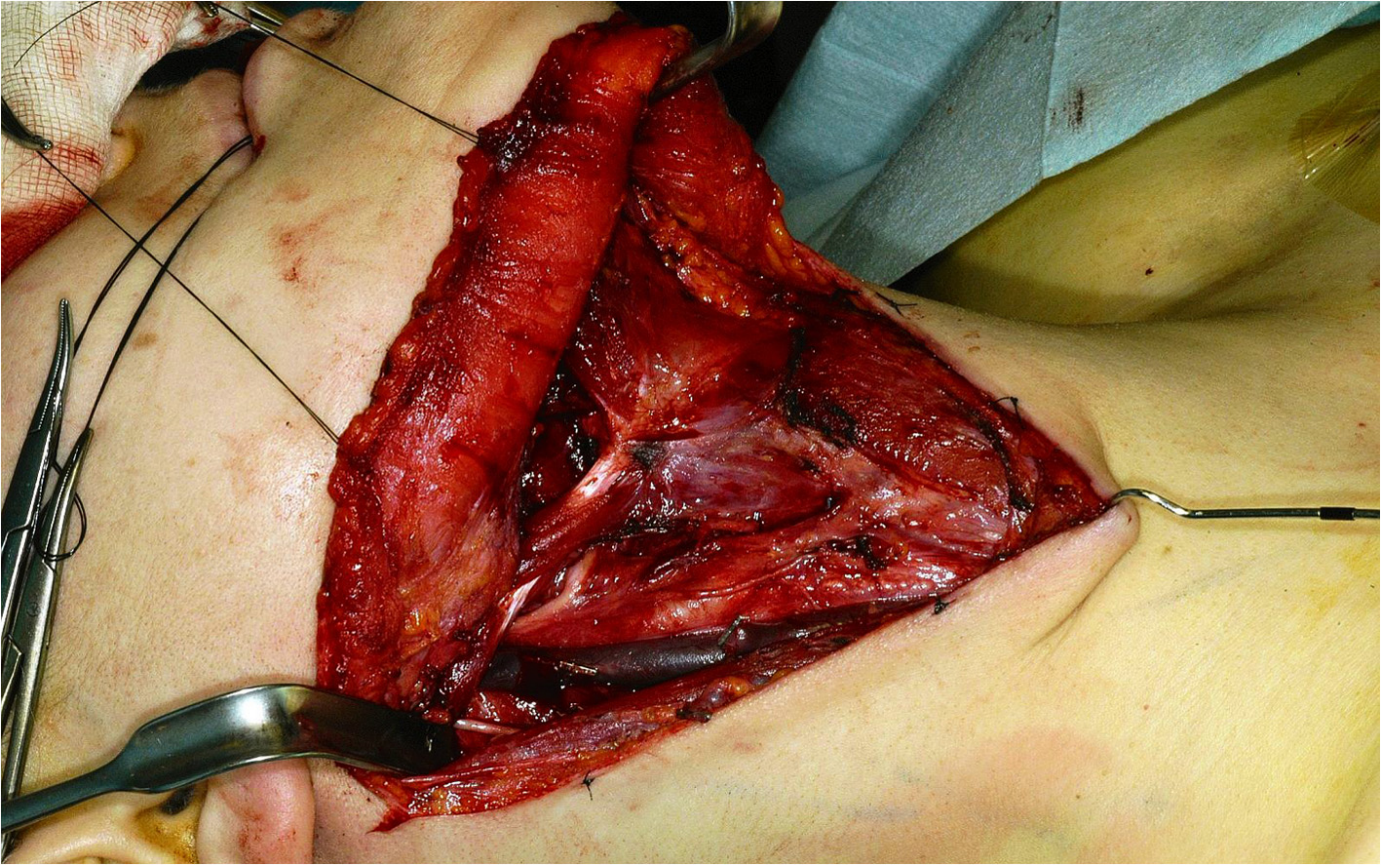
Raised radial forearm free flap.

**Figure 5:**
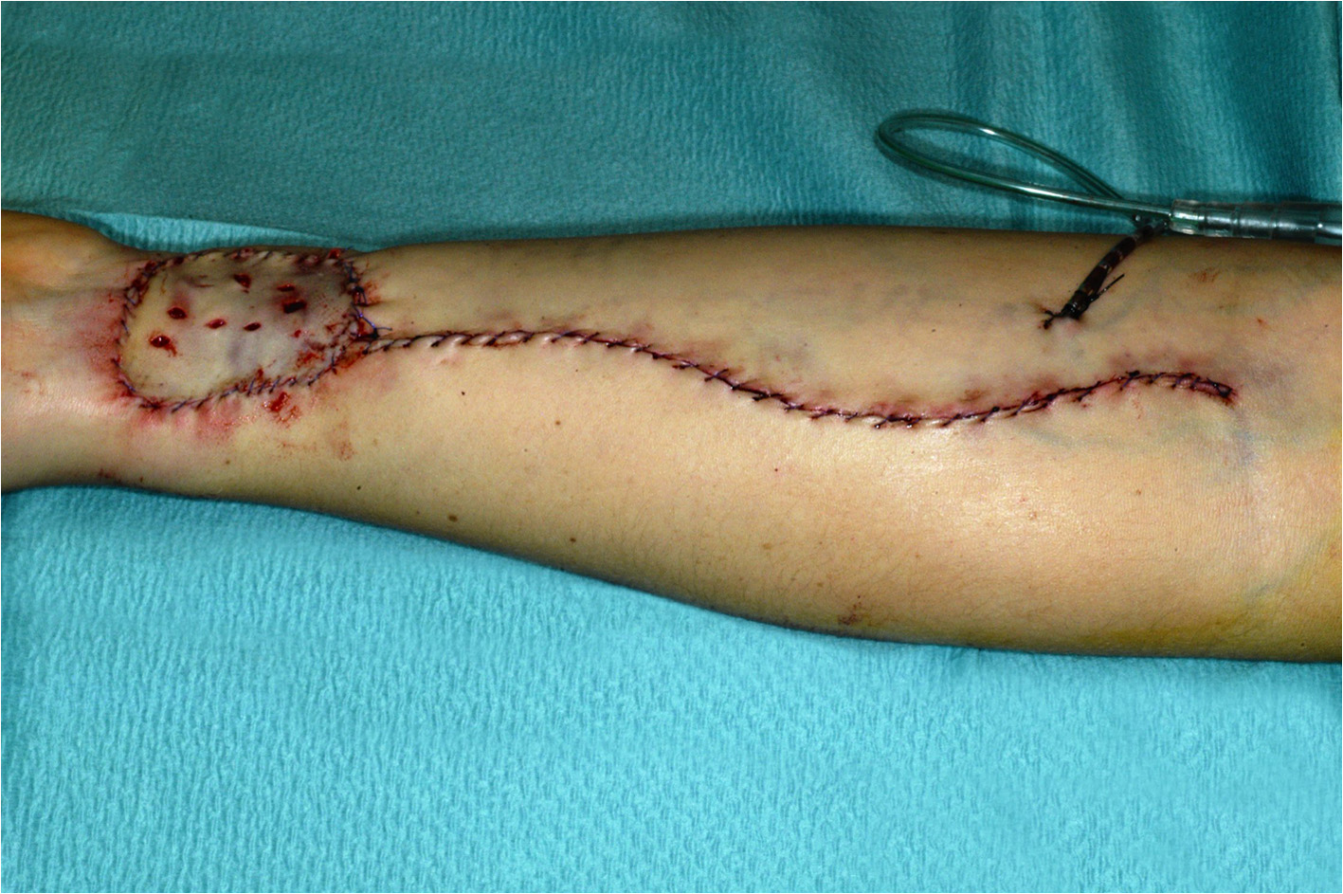
Direct post operative donor site.

**Figure 6:**
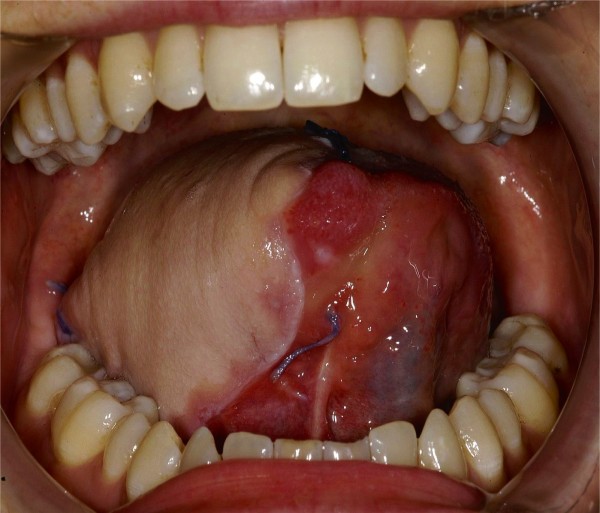
Donor site 3 weeks post operatively.

**Figure 7:**
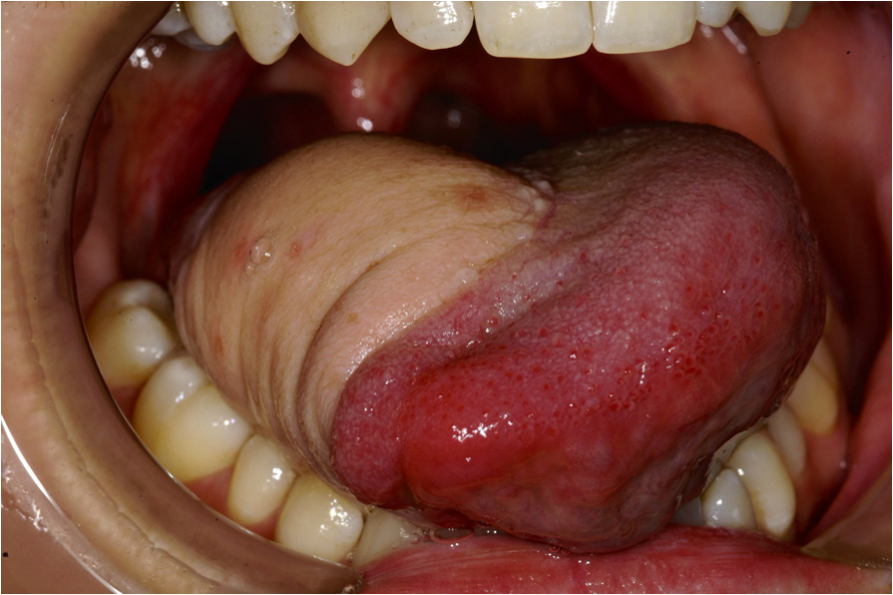
3 week post operative view showing good tongue mobility.

**Figure 8:**
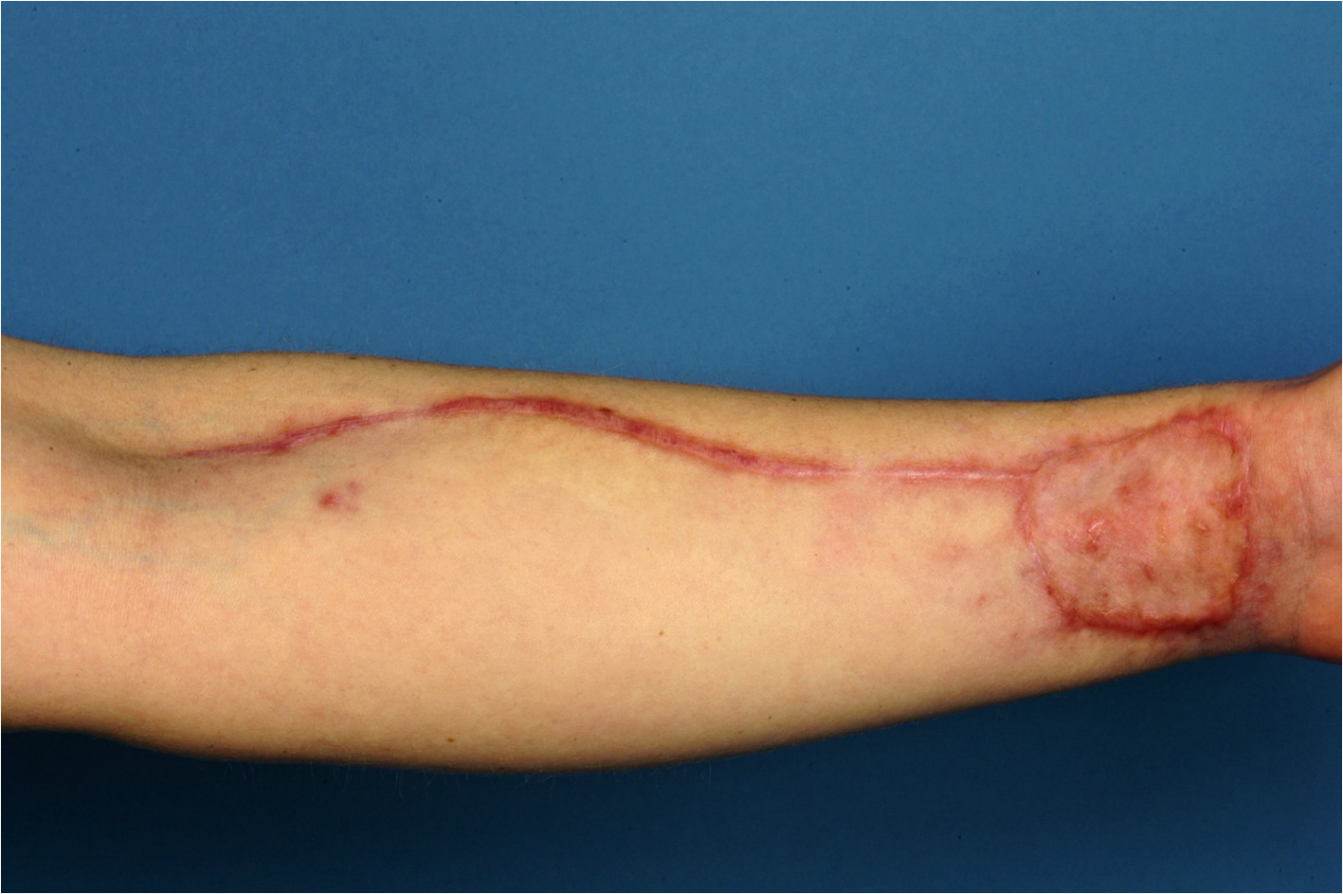
Lateral view 3 weeks post operatively.

Considering the clinical appearance and the history of the quick growth of the tongue’s lesion we postulated a malignant alteration and on the day of the first examination a biopsy under local anesthesia was performed. The histopathology showed a moderately differentiated, squamous cell carcinoma with surrounding chronic inflammation. The patient was transferred to the Cranio-Maxillofacial and Oral Surgery Division of the University Hospital Zurich. A MRI (Magnetic Resonance Imaging) as well as a PET (Positron Emission Tomography) scan was performed as per standard oncological staging. No evidence of locoregional lymphatic spread or distant metastasis could be found which led to a cT2N0M0 staging. Eight days after the first examination at the same department a partial glossectomy was conducted on the right side and neck dissection of levels I-III (Figure 3). Due to the extent of the resection a reconstruction of the tongue with a radialis free vascularised flap from left side was performed (Figures 4, 5 and 6). Full thickness skin graft from the medial side of the upper arm was used to cover the donor site (Figures 7 and 8).

The histological analysis confirmed the presence of a moderately differentiated squamous cell carcinoma of the right tongue. The surgical excision margins were all safely clear of tumor and no positive lymph nodes were noted with a final staging of pT1pN0(0/28)pR0. Due to the R0 resection, no positive lymph nodes and absence of histological risk factors no further treatment was recommended. A standardized follow up protocol was followed.

### Discussion

As the median age at diagnosis of tongue cancer is 61 years and only 2% of patients are diagnosed before the age of 35 these are thus rare occurrences and warrants further attention.

Our 26-year old patient survived acute myeloid leukemia (AML) before her second year. These improved survival rates will become more common as drug therapies improve.

The main treatment for AML is chemotherapy that usually comprises of different phases: In the first phase (induction), a combination of chemotherapy drugs is given to the patient to destroy the leukemia cells and enable the bone marrow to regain normality. The treatment is continued for a period of time even after there are no more signs of leukemia in the blood or bone marrow examinations to rule out any recurrence. This post-remission treatment usually involves two or three further cycles of chemotherapy. A bone marrow transplantation, following the standard chemotherapy, is applied only for children with AML at high risk of recurrence. AML may at times develop in the central nervous system that can be prevented by injecting chemotherapy drugs directly into the spinal fluid during a lumbar puncture. This chemotherapy is usually given after each of the two previously mentioned courses of chemotherapy. Occasionally, radiotherapy may play a role in disease affecting the brain.

Chemotherapy drugs and radiation are known for their long term carcinogenic effects; therefore second malignancies are one of the most serious side effects of treatment of childhood cancer survivors. The risk of developing a second malignancy in 20-year survivors, following a first childhood malignancy, is estimated at 3-12%, [20] this represents a 10 fold [21] or 10–20 fold [18] increase in survivors of childhood AML as compared to the general population.

On the one hand cumulative incidence of second malignant neoplasm (SMN) varies depending on the original cancer diagnosis. Assessment of second neoplasms according to the Childhood Cancer Survivor Study (CCSS), published by Neglia at al 2001, demonstrates that SMNs are significantly associated with initial cancer diagnosis of Hodgkin disease or soft tissue sarcoma.

Table 1 derived from Joseph P. Neglia 2001 [17].

**Table 1:** A cumulative incidence of second neoplasms 20 years from original cancer diagnosis

	Cumulative incidence of second malignant neoplasm at 20 y, %	Excess absolute risk/1000 person-years of follow-up
Overall	3.18	1.88
Hodgkin	7.63	5.13
Soft tissue sarcoma	3.98	2.33
Neuroblastoma	1.87	0.95
Kidney tumor	1.62	1.01
Leukemia	2.05	1.20
Bone cancer	3.28	1.79
Central neural system	2.14	1.13
Non-Hodgkin	1.87	0.89

Excess absolute risk is determined by subtracting the expected number of malignancies in the cohort from the observed number, dividing the difference by the person-years of follow-up, and multiplying by 1000.

On the other hand incidence of the SMN depends on the treatment of the primary malignancy. [22] Radiation therapy is a major cause of SMN. [23,24] Eighty to ninety percent of SMNs following the radiation therapy occurs within the radiation field. Typical SMNs after Radiotherapy are skin cancer, thyroid cancer and breast cancer (in female survivors of Hodgkin’s Lymphoma, 57- fold risk compared with the general population) [24].

In contrary to the radiotherapy associated solid tumors, chemotherapy mostly causes hematologic second malignancies. [17].

Assessment of risk according to type of second cancer demonstrated that the highest risks were seen for secondary tumors involving bone, breast, thyroid and central nervous system.

Table 2 derived from Joseph P. Neglia 2001 [17].

**Table 2:** Risk according to type of second cancer

	Standardized Incidence Ratio
Overall	6.38
Bone cancer	19.14
Breast cancer	16.18
Thyroid cancer	11.34
Central neural system	9.85
Leukemia	6.86
Soft-tissue sarcoma	6.33
Melanoma	4.04
Lymphoma	1.51
Other	4.01

According to CCSS beside second neoplasm’s other therapy - related outcomes are early death, organ dysfunction such as cardiac, pulmonary, and endocrine, gonal, decreased fertility reduced growth and impaired intellectual function.

Although the role of the chemotherapy and radiation is well established, variability between individuals exist, which suggests a role for genetic variation in the susceptibility to genotoxic exposures. This individual variability is probably related to polymorphism genes that regulate the availability of the active drug metabolite or other mechanisms responsible for DNA repair. Polymorphism in genes implicated in drug metabolism and transport are relevant for disease-free survival and drug toxicity. [25] Variation in DNA repair plays a part in susceptibility to de-novo cancer and probably modifies the risk of second primary cancers after exposure to radiation and chemotherapy [26].

In conclusion it can be stated that improvements in the treatment of malignancies of childhood thankfully leads to more survivors. This is however slightly shaded by the fact that these patients may develop a second malignancy later in life. It is conceded that this may be a multi factorial process with other risk factors like genetic predisposition, environmental exposure, hormonal and alterations in immune function playing a up to yet undefined role. By optimizing early diagnosis as well as treatment strategies the life span of these patients may be prolonged further.

## Consent

Written informed consent was obtained from the patient for publication of this report and any accompanying images

## Abbreviations

AML, acute myeloid leukemia; CCSS, Childhood Cancer Survivor Study; EBV, Epstein-Barr virus; HNSCC, head and neck squamous cell carcinoma; HPV, human papilloma virus; HSV, herpes simplex virus; IL6, interleukin 6; MRI, Magnetic Resonance Imaging; PET, Positron Emission Tomography; SCC, Squamous cell carcinoma; TNF, tumor necrosis factor.

## Competing interests

The authors declare that they have no competing interests.

## Authors’ contributions

AC: First examination of the patient, biopsy, preparation of the manuscript. ML: Reviewing of the manuscript. MB: Operation of the patient, coordinating and reviewing of the manuscript. All authors read and approved the final manuscript.
